# Single nucleotide polymorphisms in premature ovarian failure-associated genes in a Chinese Hui population

**DOI:** 10.3892/mmr.2015.3762

**Published:** 2015-05-08

**Authors:** LILI MA, YAN CHEN, SI MEI, CHUNLIAN LIU, XIAOHONG MA, YONGLI LI, YINZHI JIANG, LINGXIA HA, XIAN XU

**Affiliations:** 1 Center for Reproductive Medicine, General Hospital of Ningxia Medical University, Yinchuan, Ningxia 750004, P.R. China; 2 Key Laboratory of Fertility Conservation and Maintenance, Ministry of Education, General Hospital of Ningxia Medical University, Yinchuan, Ningxia 750004, P.R. China; 3 Key Laboratory of Reproduction and Genetics, General Hospital of Ningxia Medical University, Yinchuan, Ningxia 750004, P.R. China

**Keywords:** premature ovarian failure, growth differentiation factor 9, bone morphogenetic protein 15, inhibin βB, gene mutation, follicle stimulating hormone, Ningxia Hui

## Abstract

Premature ovarian failure (POF) is an ovarian defect characterized by the premature depletion of ovarian follicles in individuals <40 years old, and is a major cause of infertility in females. Genetic factors are considered to be responsible for the development of POF, however, the exact pathogenesis remains to be elucidated in the majority of cases. In the present study, the single nucleotide polymorphisms (SNPs) of growth differentiation factor 9 (GDF9), bone morphogenetic protein 15 (BMP15), inhibin βB (INHBB) and follicle stimulating hormone receptor (FSHR) genes were investigated, and their association with POF in a Chinese Hui population of the Ningxia Hui Autonomous Region in western China was evaluated. Peripheral blood samples were collected from 63 patients diagnosed with POF (POF group) and 58 normal control individuals (control group), from which the genomic DNA was isolated. The *GDF9*, *BMP15*, *INHBB* and *FSHR* genes were amplified using polymerase chain reaction assays, and their SNPs were determined by sequencing. In the four SNPs identified across the *GDF9* loci, D57Y (169G>T), rs1049127 (546G>A), rs254286 (447C>T) and rs254285 (969C>G), the frequencies of the 546G>A genotype and allele A were significantly higher in the POF group, compared with the normal control group (34.92, vs. 6.90%; P<0.05 and 19.05, vs. 3.23%; P<0.05, repsectively), while no significant differences were observed in the occur rence of the c.447C>T and c.969C>G mutations between the two groups (60.32, vs. 50% and 50.79, vs. 55.17%, repsectively). The c.169G>T mutation within the *GDF9* gene was only detected in two patients with POF, and the mutation did not occur in the normal control group. A total of three SNPs were detected within the *BMP15* gene, including rs3810682 (−9C>G), rs79377927 (788_789insTCT) and rs17003221 (852C>T), and no significant differences were observed in the frequencies of the 9C>G and 852C>T genotypes between the POF and control groups (7.94, vs. 6.90% and 4.76, vs. 3.45%, respectively). The 788_789insTCT genotype was detected in only two patients with POF. A novel mutation, c.1095C>A, was identified in exon 2 of the *INHBB* gene, however, no significant difference was found in the occurrence of the mutation between the two groups (30.16, vs. 22.41%; P>0.05). The rs6165 (919G>A) and rs6166 (2039G>A) SNPs were detected in exon 10 of the *FSHR* gene; however, no significant difference was observed in the genotype frequencies between the two groups (92.06, vs. 91.38% and 96.83, vs. 93.10%, respecrively). These results demonstrated that *GDF9* c.169G>T (D57Y), c.546G>A (rs1049127), and *BMP15* rs79377927 (788_789insTCT) were associated with POF in the Chinese Hui population.

## Introduction

Premature ovarian failure (POF) is defined as the occurrence of amenorrhea, hypergonadotropinemia and estrogen deficiency in women <40 years of age, which is accompanied by decreased levels of estrogen and increased levels of gonado tropin (1,2). It is a major cause of female infertility (3,4) and it has been reported that females diagnosed with POF have almost a two-fold age-specific increase in mortality rate, and are at increased risk of cardiovascular disease, neurocognitive disorders, including Parkinson’s disease, and endocrine and autoimmune disorders (5–13). Notably, the early loss of ovarian function has significant psychosocial sequelae and health implications (5). Although often considered a rare disorder, POF affects 0.0001% of femals by the age of 20 years, 0.001% by 30 years and 0.01% by 40 years (14). In addition, the relevance of POF is continuously increasing as females are tending to conceive more frequently in their thirties and forties. A previous multi ethnic population, cross sectional study revealed that the prevalence of POF was 1.0% in Caucasian females, 1.4% in African American females, 1.4% in Hispanic females, 0.5% in Chinese females and 0.1% in Japanese females, suggesting significant differences in the frequency of POF among ethnic groups (P=0.01) (15). In China, the incidence of POF is estimated to be 1–3.8% in females aged <40 years (16).

A number of hypotheses have been suggested to explain the development of POF (17). It has been reported that POF can be induced by chemotherapy, radiotherapy, autoimmune disorders, ovarian or other pelvic surgery, and genetic disorders, including Turner syndrome and Fragile X syndrome (18). In addition, POI has been revealed to have a significant genetic component (19). Although the underlying cause of POF remains to be elucidated in the majority of cases, mutations in certain candidate genes have been reported to be associated with the risk of POF (20–26). In the present study, single nucleotide polymorphisms (SNPs) of the growth differentiation factor 9 (GDF9), bone morphogenetic protein 15 (BMP15), inhibin βB (INHBB) and follicle stimulating hormone receptor (FSHR) genes were investigated in a Chinese Hui population in Ningxia, western China, and their association with POF was evaluated.

## Patients and methods

### Patients

Patients of Chinese Hui ethnicity, with a diagnosis of POF were recruited from the Center for Reproductive Medicine, the General Hospital of Ningxia Medical University (Yinchuan, China) during the period between February 2010 and October 2011 for investigation in the present study. All participants met the following inclusion criteria: i) ≤40 years old; ii) duration of amenorrhea ≥6 months; iii) serum FSH level ≥40 IU/l or serum luteinizing hormone (LH) level ≥30 IU/l on two or more occasions; iv) serum estradiol (E_2_) level ≤25 pg/ml in two occasions in two consecutive months, with the presence of amenorrhea; v) B mode ultrasound indicating no follicle reserves in either ovary; vi) no autoimmune diseases, endocrine, liver or kidney dysfunctions, or impaired glucose tolerance. Individuals with a history of ovarian surgery, radiotherapy, chemotherapy or other factors, which may damage ovarian functions were excluded. A total of 63 patients were enrolled in the present study, among which two were biological sisters, with normal karyotypes. The patients had a mean age of 29.82±6.0 years (range, 17–39 years).

A total of 58 women of childbearing age of the Chinese Hui population, who were admitted to the Center for Reproductive Medicine, General Hospital of Ningxia Medical University due to tubal factor or male factor infertility over the same period, were recruited as controls. These individuals had a mean age of 29.25±4.43 years (range, 22–41 years). All the control individuals had a regular menstrual cycle, and normal reproductive hormone levels and chromosomes. Transvaginal B mode sonography (Aloka SSD 1400 Ultrasound system; Hitachi Aloka Medical, Ltd., Tokyo, Japan) of the uterus and bilateral annex revealed no organic diseases, and the ovarian antral follicles were normal.

### Ethical considerations

The present study was approved by the Ethics Review Committee of the General Hospital of Ningxia Medical University (permission no. NZ-IRB-2010017). Written informed consent was obtained from all participants following a detailed description of the potential benefits of the investigation.

### DNA extraction, polymerase chain reaction (PCR) assay and sequencing

Following overnight fasting, a 5 ml venous blood sample was collected from the elbow, anticoagulated with EDTA Na_2_ (Amresco, LLC, Solon, OH, USA) and stored at −80°C for the subsequent experiments. Genomic DNA was extracted from the blood using a TIANamp genomic DNA kit (Tiangen Biotech, Beijing, China), according to the manufacturer’s instructions, and the concentration was determined using ultraviolet spectrophotometry (UV3100; Hitachi, Ltd., Tokyo, Japan). PCR amplification of the *GDF9*, *BMP15*, *FSHR* and *INHBB* genes was performed using the primers shown in Table I, in a 25 *μ*l reaction volume containing 1 *μ*l DNA template, 12.5 *μ*l PCR mix, 0.75 *μ*l forward and reverse primers, 0.25 *μ*l Taq DNA polymerase and 10.5 *μ*l ddH_2_O, under the PCR amplification conditions shown in Table I. The PCR was conducted on an ABI 3100 sequencer (Applied Biosystems, Foster City, CA, USA). The PCR products were confirmed using gel electrophoresis on a 2% agarose gel (Sangon Biotech Co., Ltd., Shanghai, China). All PCR reagents, primers and kits were purchased from Sangon Biotech Co., Ltd. Subsequently, the PCR products were sequenced for *GDF9*, the *BMP15* gene protein coding region, *INHBB* gene *exon 2* and the *FSHR Ala307Thr* and *Ser680Asn* variants at the Beijing Genomics Institute (Beijing, China) using a dideoxy chain termination method (27).

### Statistical analysis

The sequencing chromatograms were assessed using the Chromas 2.23 program (Technelysium, Tewantin, QLD, Australia), and the sequences of the *GDF9*, *BMP15*, *INHBB* and *FSHR* genes were aligned to those registered in GenBank (http://www.ncbi.nlm.nih.gov/genbank/) for the identification of mutatnt loci. All data were entered into Microsoft Excel 2007 (Microsoft Corporation; Redmond, WA, USA), and all statistical analyses were performed using SPSS version 17.0 statistical software (SPSS, Inc., Chicago, IL, USA). The differences in proportions were assessed for statistical significance using a χ^2^ test. P<0.05 was considered to indicate a statistically significant difference.

## Results

### GDF9 SNPs

**Figure 1:**
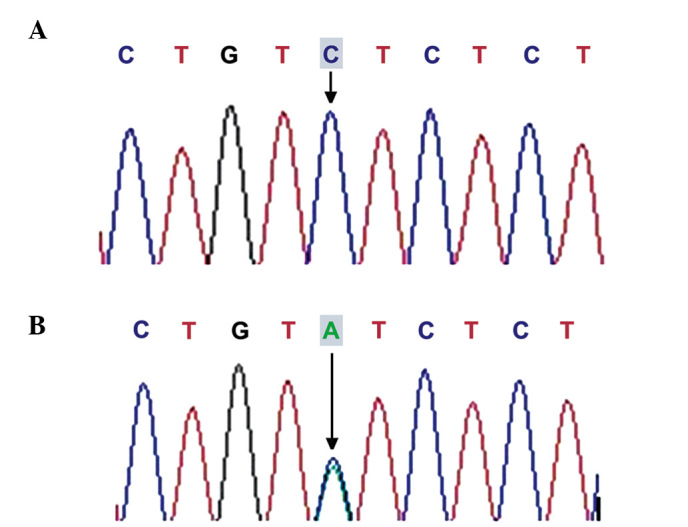
Sequencing map of *GDF9* 169G>T. (A) GG wild type genotype; (B) GT heterozygote. GDF9, growth differentiation factor 9.

**Figure 2:**
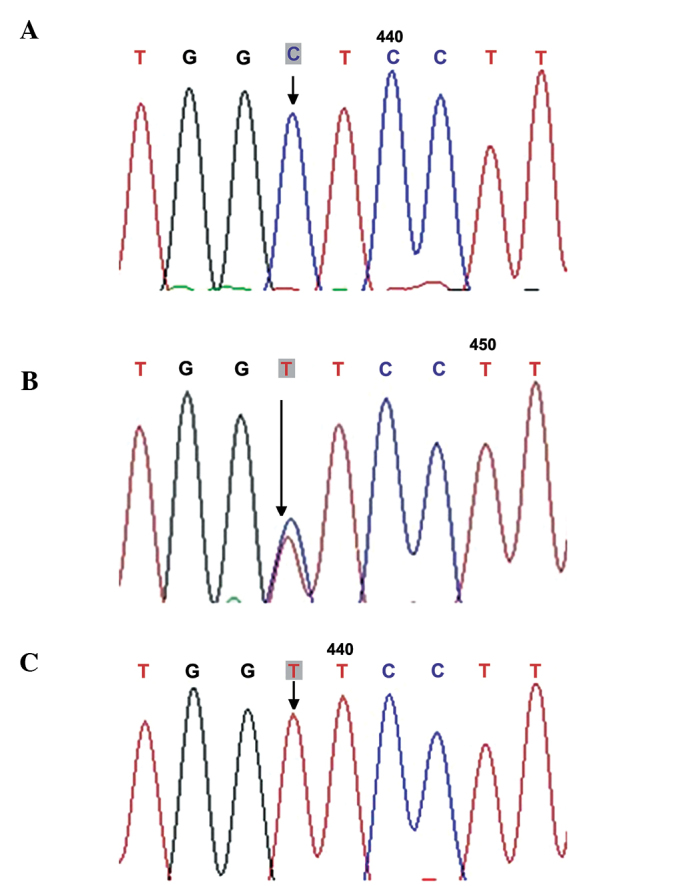
Sequencing map of the 546G>T growth differentiation factor 9 gene mutation. (A) GG wild type genotype; (B) GT heterozygote; (C) TT homozygous mutation.

**Figure 3:**
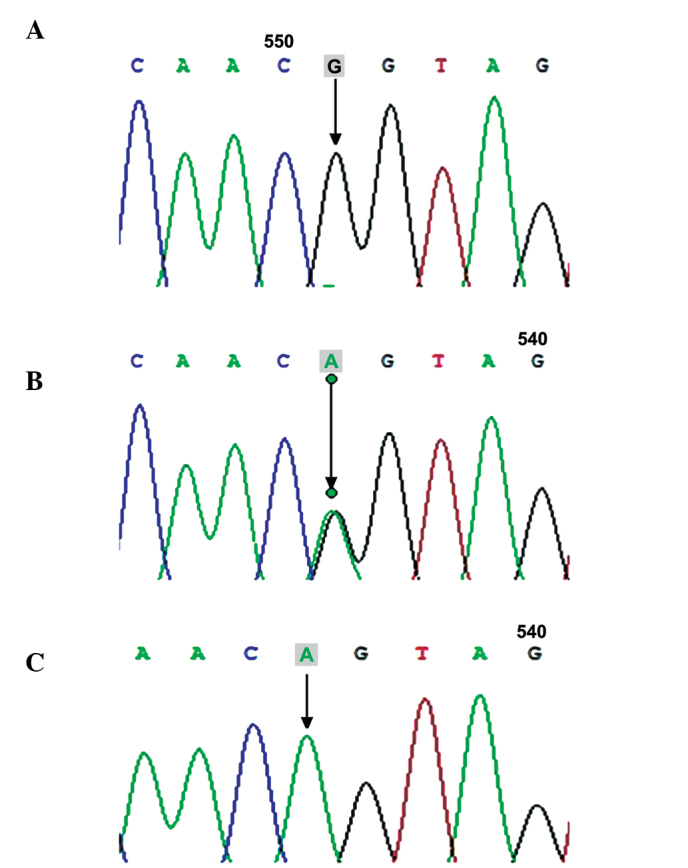
Sequencing map of the 448C>T growth differentiation factor 9 mutation. (A) CC wild type genotype; (B) CT heterozygote; (C) TT homozygous mutation. GDF9, growth differentiation factor 9.

**Figure 4:**
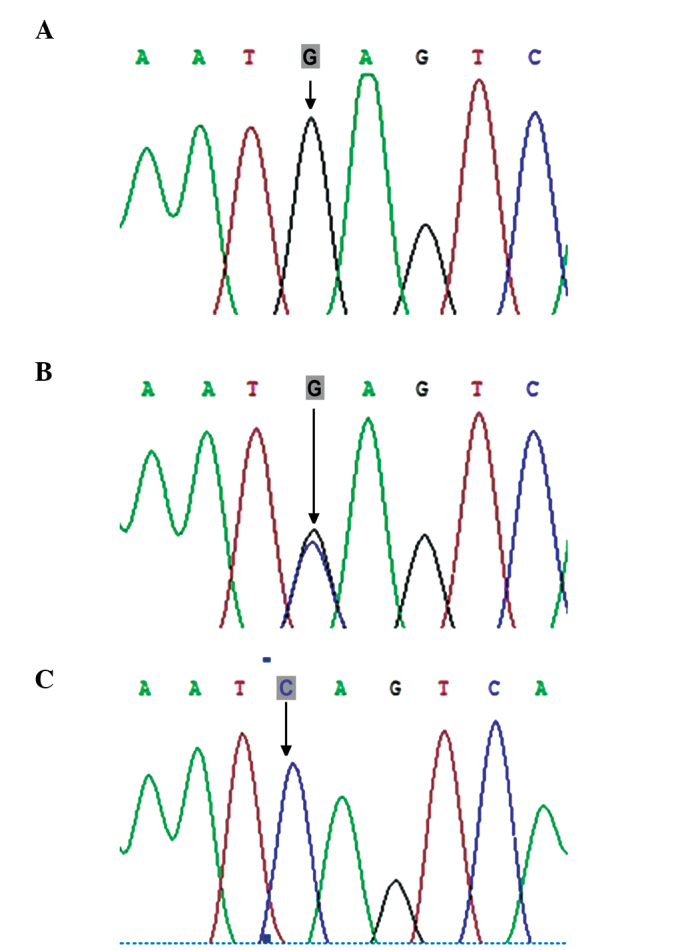
Sequencing map of *GDF9* 969C>G. (A) CC wild type genotype; (B) CG heterozygote; (C) GG homozygous mutation. GDF9, growth differentiation factor 9.

A total of four SNPs were genotyped across the *GDF9* locus using sequencing analysis, including D57Y (169G>T; Fig. 1), rs1049127 (546G>A; Fig. 2), rs254286 (447C>T; Fig. 3) and rs254285 (969C>G; Fig. 4). The c.169G>T (D57Y) missense mutation has not been detected previously, and occurred in two patients with POF, while the mutation was not detected in the control individuals. The frequencies of the 546G>A genotype and allele A were significantly higher in the POF group than in the normal control group (34.92, vs. 6.90% and 19.05, vs. 3.23%, respectively; P<0.05), while no significant differences were observed in the frequencies of the c.447C>T and c.969C>G mutations between the two groups (60.32, vs. 50% and 50.79, vs. 55.17%; P>0.05; Table II).

### BMP15 SNPs

**Figure 5:**
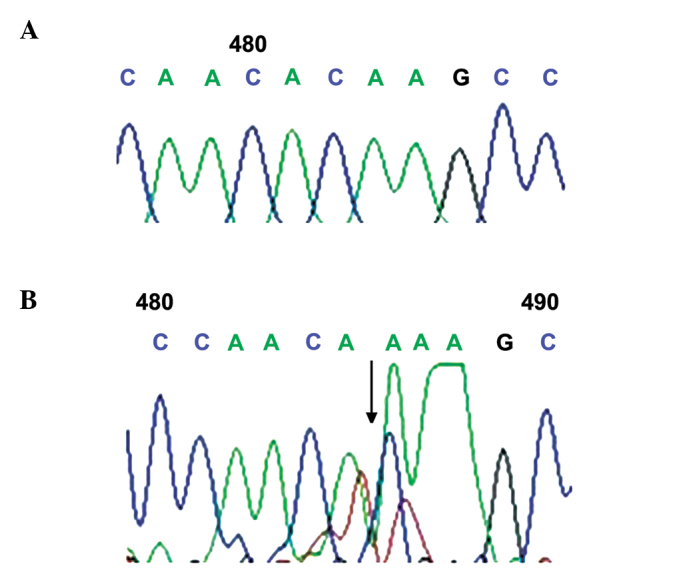
Sequencing map of the rs79377927 (788_789insTCT) bone morphogenetic protein 15. (A) wild type genotype; (B) insTCT.

**Figure 6:**
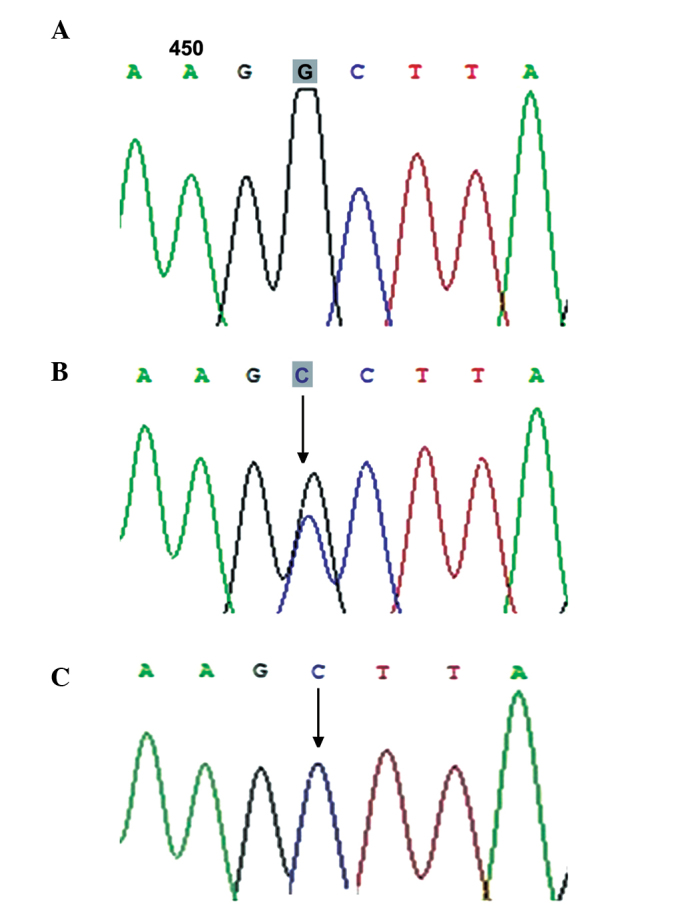
Sequencing map of the −9C>G bone morphogenetic protein 15 mutation. (A) CC wild type genotype; (B) CG heterozygote; (C) GG homozygous mutation.

**Figure 7:**
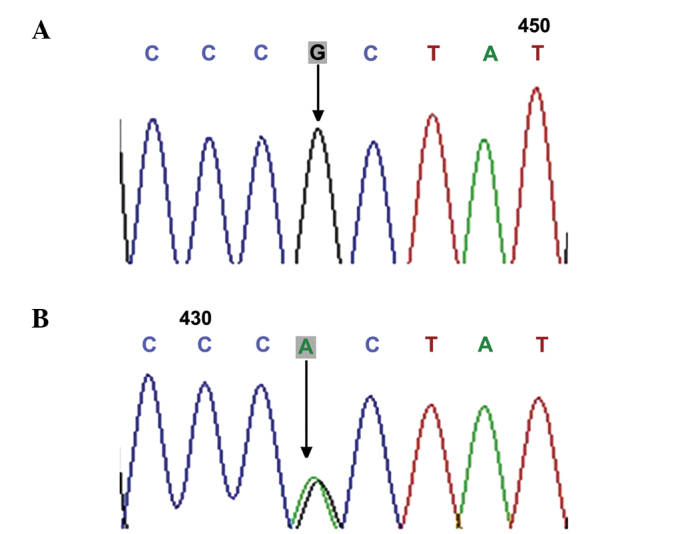
Sequencing map of the 852C>T bone morphogenetic protein 15 mutation. (A) CC wild type genotype; (B) CT heterozygote.

The present study detected three SNPs within the *BMP15* gene, including rs79377927 (788_789insTCT; Fig. 5), rs3810682 (−9C>G; Fig. 6), and rs17003221 (852C>T; Fig. 7), and the frequencies of the 9C>G and 852C>T genotypes did not vary significantly between the POF and control groups (7.94, vs. 6.90% and 4.76, vs. 3.45%; P>0.05), while the 788_789insTCT genotype was detected in two patients with POF (Table II).

### INHBB SNPs

**Figure 8:**
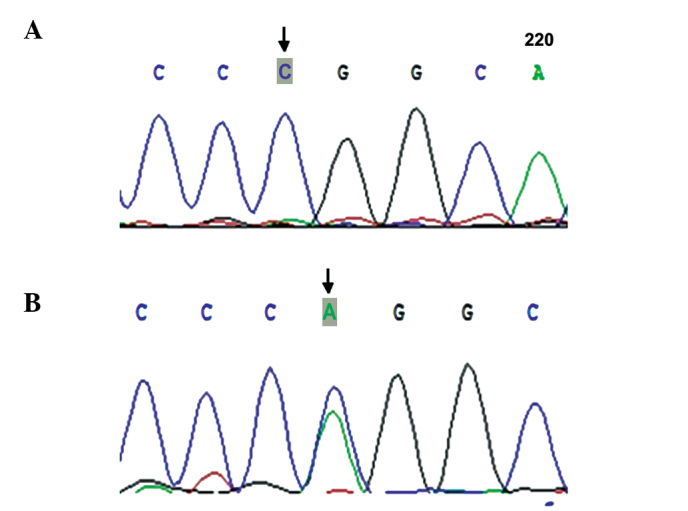
Sequencing map of the 1095C>A inhibin B mutation. (A) CC wild type genotype; (B) CA heterozygote.

Exon 2 of the *INHBB* gene was sequenced and the novel synonymous mutation locus Por365Por (Fig. 8) was identified. However, no significant difference was observed in the occurrence of the mutation between the POF and control groups (30.16, vs. 22.41; P>0.05; Tables II and III).

### FSHR SNPs

**Figure 9:**
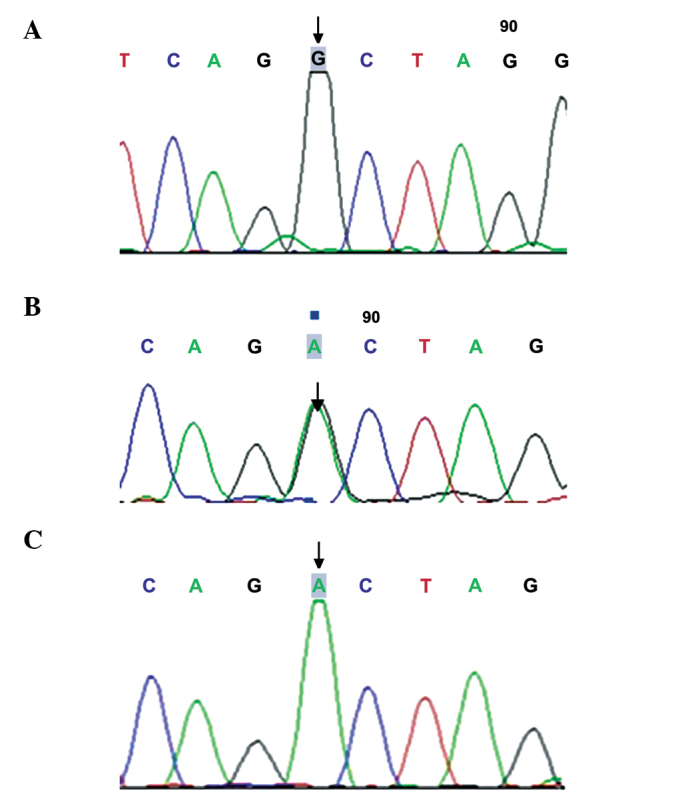
Sequencing map of the 919G>A follicle stimulating hormone receptor gene. (A) GG wild type genotype; (B) GA heterozygote; (C) AA homozygous mutation.

**Figure 10:**
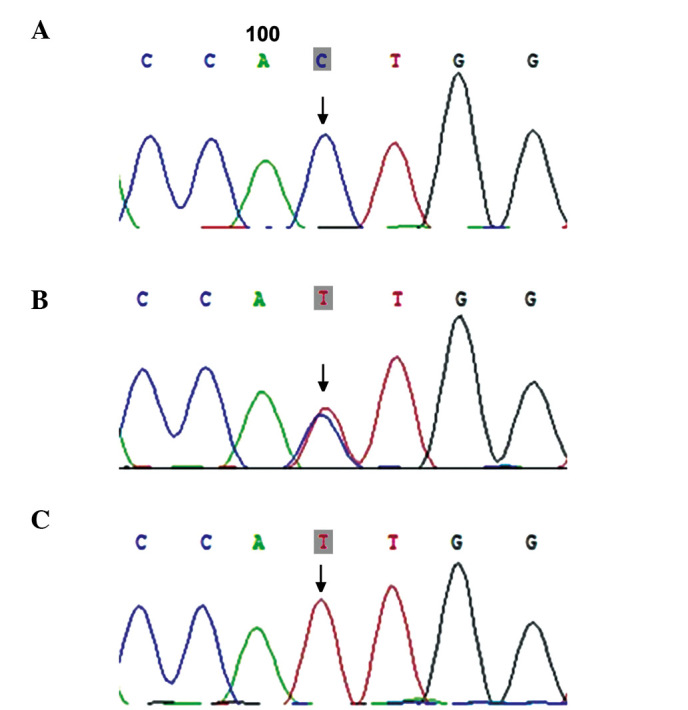
Sequencing map of the 2039G>A follicle stimulating hormone receptor.gene mutation. (A) GG wild type genotype; (B) GA heterozygote; (C) AA homozygous mutation.

A total of two SNPs of rs6165 (919G>A) and rs6166 (2039G>A) were detected in exon 10 of the *FSHR* gene (Figs. 9 and 10); however, the frequency of these two mutations exhibited no significant difference between the POF and control groups (92.06, vs. 91.38% and 96.83, vs. 93.10%; P>0.05; Table II). In addition, no significant differences were observed in the genotype frequency of the FSHR gene between the two groups (P>0.05; Table III).

Analysis of the interaction between the *FSHR* SNPs and *GDF9* SNPs revealed no significant differences in the frequencies of the *FSHR* 307-*GDF9* 546, *FSHR* 680-*GDF* 546 and *FSHR* 307-680 combined genotypes between the POF and control groupa (P>0.05; Tables IV–VI).

## Discussion

*GDF9*, a member of the transforming growth factor-β superfamily, is expressed in oocytes and is considered to be a requirement for ovarian folliculogenesis (28). *GDF9* mutations have been associated with POF (29). Dixit *et al* (30) reported that two rare missense mutations, c.199A>C and c.646G>A, in Indian females with ovarian failure, were associated with ovarian failure, and the presence of the c.447>T mutation was considered to infer a higher risk for POF. In 203 patients with POF, the heterozygous transversion, 557C>A, in exon 2 within the *GDF9* gene was associated with POF (31). Zhao *et al* (32) identified four SNPs across the *GDF9* coding regions in 100 Chinese females with POF, including c.436C>T (p.Arg146Cys), c.588A>C (silent), c.712A>G (p.Thr238Ala), and c.1283G>C (p.Ser428Thr), and the nonsynonymous SNPs c.436C>T and c.1283G>C were also detected in 96 control individuals. The c.712A>G perturbation, which results in a missense mutation (p.Thr238Ala), was not detected in any of the control individuals.

In the present study, a novel missense mutation, c.169G>T (D57Y), was detected in the *GDF9* gene, which was present in two patients with POF and was not detected in the control individuals. This mutation was present in the proprotein region of the *GDF9* gene, and the G T mutation at position 169 led to the substitution of aspartic acid with glutamic acid at position 57. To the best of our knowledge, this is the first time that this mutant locus has been observed in patients with POF. Previously, the *GDF9* G169A was identified in Chinese females with a diminished ovarian reserve, without abnormal protein secretion or activity, which may contribute to the location of this mutant locus in the non conserved sequence of the GDF9 protein (33).

The c.546G>A (p.Glu182Glu) mutation occurs at exon 2 of the *GDF9* gene, which was initially identified in Indian females with ovarian failure (30). Laissue *et al* (31) reported frequen cies of 23.15% (47/203) and 54.26% (14/54) of the 546G>A mutation in French patients with POF and normal controls, respectively, and this 546G>A variation was considered to be a common synonymous mutation in the French population. Zhao *et al* (32) detected the c.546G>A heterozygous point mutation in 26 of 100 patients with POF (26%) and 28 of 96 control individuals (29.17%), with no significant differences observed. In the present study, the frequency of the c.546G>A mutation was significantly higher in the patients with POF (34.92%), compared with the control individuals (6.90%; P<0.05), indicating that the c.546G>A mutation was closely associated with POF in the Chinese Hui population.

In India, a case-control study revealed an 85.04% genotype distribution of c.447C>T in females with POF, which was higher than that in control individuals (χ^2^=5.93; P=0.05) (30). In 203 women with POF, recruited from several clinical centers in France, the frequency of c.447C>T was 75.9% in patients with POF and 55.5% in control individuals (31). Considering these findings, the c.447C>T mutation was hypothesized to correlate with a high risk of POF. In the present study, the frequency of c.447C>T was higher in the patients with POF than in the control individuals (60.32, vs. 50%, respectively), however, no significant difference was observed (P>0.05). This finding was consistent with previous investigations in Indian and French populations (30,31). In total, four SNPs were identified in the *GDF9* gene in patients of the Chinese Hui population with POF, and the c.169G>T and c.546G>A mutations within the *GDF9* gene were found to closely correlate with the development of POF in these patients.

As a paracrine signaling molecule involved in oocyte and follicular development, the *BMP15* gene is considered to be one of the important candidate genes involved in POF (33). However, various incidence rates of the *BMP15* mutation have been detected in patients with POF of different ethnicities (34), and the correlation between the *BMP15* mutation and POF development varies among them (35–37). The c.985C>T missense mutation in the *BMP15* gene, which was initially identified in Chinese females with POF in 2010 (38), has been found to result in alteration of the polar/positive amino acid, arginine, to the nonpolar and neutral amino acid, cysteine, which is located in the region of biologically active *BMP15*, and this c.985C>T variant has been reported to be an important potential disease associated mutation in *BMP15* among patients with POF (38). However, Zhang *et al* (39) demonstrated rare mutations in BMP15 exons, and changes in the *BMP15* pro-peptide in Chinese females with POF, and concluded that the two SNPs rs17003221 (CT) in exon 2 and rs (3810682CG: ss16336587) in the putative promoter region of exon 1 were not associated with POF. In the present study, three SNPs were identified in the coding region of the *BMP15* gene, rs3810682 (−9C>G) and rs79377927 (788_789insTCT) in exon 1 and rs17003221 (852C>T) in exon 2, which were detected in patients with POF and the control individuals. The findings revealed a significantly higher frequency of allele C, compared with allele G in the SNP-9C>G at exon 1 of the BMP15 gene (95.97, vs. 4.03%; P<0.05), which was consistent with a previous study (38). This further demonstrated that, unlike European populations and other Asian populations, the C allele was predominant in SNP-9C>G within the BMP15 gene. No missense mutations, associated with the development of POF, were identified in the coding region of *BMP15*. As the coding region of the BMP15 protein is highly conserved across species (40), it was hypothesized that BMP15, although involved in the pathogenesis of POF, may not be the major factor causing diminished ovarian function, which may be explained by other specific mechanisms, including disorders of pre-translation processing, including mRNA splicing.

INH is a dimeric glycoprotein composed of an α-subunit (INHA) and one of two possible β-subunits (INHBA or INHBB), which has been defined as a gonadal hormone that exerts a specific negative feedback action on the secretion of FSH from the gonadotropic cells of the pituitary gland (41,42). It has been reported that *INH* is a candidate gene for the development of POF (43), and the *INHA* gene has been found to closely correlate with POF (44–46), while the *INHBB* and *INHBA* genes are not associated with ovarian failure (47). In the present study, a synonymous mutation (c.1095C>A) at exon 2 of the *INHBB* gene was identified. Generally, this mutation does not affect the original amino acid sequence or the carrier’s ovarian function, however, the present study reported a higher frequency of the *INHBB* mutation in the patients with POF, compared with the controls (30.16, vs. 22.41%, respectively; P>0.05), suggesting that the *INHBB* mutation may be involved in the alteration of ovarian function and ovarian reserve.

At present, the *FSHR* gene variants reported to be associated with POF include Ala189Val, Asn191Ile, Tle160Thr/Arg573Cys, Asp224Val/leu601Val and Pro348Arg polymorphisms at exon 7 and a Phe591Ser polymorphism at exon 10 (48). In Brazilian females, the presence of the Ala307Thr polymorphism is likely to be associated with a more precocious onset of POF (49). In Argentinian females, mutations in the *FSHR* gene are rare in females with POF, and the presence of a particular *FSHR* isoform does not appear to be associated with POF (50). Whitney *et al* (51) reported that *FSHR* gene deletions were uncommon in females with POF, although the gene is polymorphic. In addition, no C566T mutation of the *FSHR* gene has been detected in patients with POF and control individuals, which appears rare in Chinese females with POF (52). In the present study, two SNPs of rs6165 (919G>A) and rs6166 (2039G>A) were detected in the *FSHR* gene; however, the frequency of these two mutations exhibited no significant difference between the POF group and the control group, and no significant differences were observed in the genotype frequency of the *FSHR* gene between the two groups.

In addition, analysis of the interaction between the *FSHR* SNPs and *GDF9* SNPs revealed no significant differences in the frequencies of the *FSHR* 307-*GDF9* 546, *FSHR* 680-*GDF* 546 or *FSHR* 307-680 combined genotypes between the POF group and the control group (all P>0.05). This may have been caused by the small sample size used in the present study, which cannot accurately reflect the interaction between the *FSHR* SNPs and *GDF9* SNPs. Therefore, further investigations with larger sample sizes are required to examine the *FSHR*-*GDF9* interactions in females with POF.

In conclusion, *GDF9* c.169G>T (D57Y) and c.546G>A (rs1049127), and *BMP15* rs79377927 (788_789insTCT) were found to be associated with POF in patients of the Chinese Hui population. The results of the present study identified regional variation in *GDF9* and *BMP15* SNPs in POF, however the underlying mechanisms require further analysis. The correlation between *GDF9* and *FSHR* genes was not detected in the present study, therefore further studies with larger sample sizes are required to comprehensively evaluate the interaction between POF and its associated genes. Screening of known mutation locis is of great significance when investigating the genetic mechanisms of molecules associated with POF. In conclusion, exogenous oocyte derived cell growth factors may be used to specifically rectify abnormal sites, and to improve the quality of life, delay the age of menopause, and increase ovarian reserve function in patients with POF.

## Figures and Tables

**Table I:** Primers and protocols used for PCR amplification of *GDF9*, *BMP15*, *FSHR* and *INHβB*.

Gene	Location	Primer sequence	PCR amplification protocol
*GDF9*	Exon 1	F, 5′-TAGTCCACCCACACACCTGA-3′; R, 5′-CCAAAAGCTTGGTGGAACAG-3′	94°C for 4 min, followed by 30 cycles at 94°C for 60 sec, 58°C for 45 sec and 72°C for 50 sec, then finally 72°C for 7 min
	Exon 2	F, 5′-CCAGTAAGGTTGCTGGGAAT-3′; R, 5′-TCCCCCACTAAATGATCAGC-3′	94°C for 4 min, followed by 30 cycles at 94°C for 60 sec, 58°C for 45 sec and 72°C for 50 sec, then finally 72°C for 7 min
*BMP15*	Exon 1	F, 5′-CTCGTTCGCTCGCCTGGCGC-3′; R, 5′-GAAGTTGCGCATCATGCTGT-3′	94°C for 4 min, followed by 30 cycles at 94°C for 60 sec, 58°C for 45 sec and 72°C for 50 sec, then finally 72°C for 7 min
	Exon 2	F, 5′-ATGCGCTTCTCCAGCTTTGT-3′; R, 5′-TATGGAGCACACCTCACCTG-3′	94°C for 4 min, followed by 30 cycles at 94°C for 60 sec, 58°C for 45 sec and 72°C for 50 sec, then finally 72°C for 7 min
*FSHR*	Exon 10	F, 5′-GAGTGTCACGCCTTCTCCTC-3′; R, 5′-GAGTGTCACGCCTTCTCCTT-3′	94°C for 4 min, followed by 15 cycles at 94°C for 30 sec, 62°C for 30 sec and 72°C for 30 sec, then finally 72°C for 8 min
*INHBB*	Exon 2	F, 5′-TTTCCGATCAGTGGCCACGC-3′; R, 5′-AAGATGGTTTCCCCAGTGAC-3′	94°C for 4 min, followed by 15 cycles at 94°C for 30 sec, 62°C for 30 sec and 72°C for 30 sec, then finally 72°C for 8 min

GDF9, growth differentiation factor 9; BMP15, bone morphogenetic protein 15; INHBB, inhibin βB; FSHR, follicle stimulating hormone receptor; PCR, polymerase chain reaction; F, forward; R, reverse.

**Table II:** Mutations of *GDF9*, *BMP15* and *INHβB* in patients with POF and control individuals.

Gene	Mutation	Exon	Sequence variation	Amino acid variation	Mutation rate (%)		P-value
					POF (n=63)	Control (n=58)	
*GDF9*	novel	1	c.169G>T	p.Asp57Tyr	3.17	–	–
	rs10491279	2	c.546G>A	p.Glu182Glu	34.92	6.90	<0.05
	rs254286	2	c.447C>T	p.Thr149Thr	60.32	50	>0.05
	rs254285	1	c.969C>G	silent	50.79	55.17	>0.05
	rs3810682	1	c.−9C>G	silent	7.94	6.90	>0.05
*BMP15*	common	1	788insTCT	262insLeu	3.17	–	–
	rs17003221	2	c.852C>T	p.Ser284Ser	4.76	3.45	>0.05
	novel	2	c.1095C>A	p.Por 365Por	30.16	22.41	>0.05
*INHBB*	rs6165	10	c.919 G>A	p.Ala307Thr	92.06	91.38	>0.05
*FSHR*	rs6166	10	c.2039 G>A	p.Ser680Asn	96.83	93.10	>0.05

GDF9, growth differentiation factor 9; BMP15, bone morphogenetic protein 15; *INHBB*, inhibin βB; FSHR, follicle stimulating hormone receptor; POF, premature ovarian failure.

**Table III:** Comparison of genotype frequency of *GDF9*, *FSHR*, and *INHβB* between patients with POF and controls.

Gene	Codon	Genotype	Frequency (%)		OR (95% CI)	χ^2^-value	P-value
			POF (n=63)	Control (n=58)			
*GDF9*	546	G/G	65.08	93.10	0.138 (0.044–0.432)	14.058	<0.05
		G/A	31.75	6.90	6.279 (1.997–19.748)	11.728	<0.05
		A/A	3.17	0	–	–	–
		G	80.95	96.77	0.152 (0.051–0.452)	14.364	<0.05
		A	19.05	3.23	6.588 (2.211–19.634)	14.364	<0.05
	447	C/C	39.68	50.00	0.658 (0.320–1.353)	1.301	>0.05
		C/T	47.62	46.55	1.044 (0.511–2.133)	0.014	>0.05
		T/T	12.70	3.45	4.073 (0.828–20.042)	3.408	>0.05
		C	63.49	75.00	0.634 (0.367–1.097)	2.665	>0.05
		T	36.51	25.00	1.572 (0.911–2.728)	2.665	>0.05
	969	C/C	47.61	44.83	1.154 (0.563–2.366)	0.153	>0.05
		C/G	39.68	43.10	0.892 (0.431–1.844)	0.095	>0.05
		G/G	11.11	12.07	0.927 (0.304–2.827)	0.018	>0.05
		C	69.05	68.55	1.104 (0.643–1.895)	0.129	>0.05
		G	30.95	31.45	0.906 (0.528–1.555)	0.129	>0.05
*FSHR*	307	G/G	7.94	8.62	0.914 (0.250–3.334)	0.019	>0.05
		G/A	39.68	43.10	0.868 (0.421–1.792)	0.146	>0.05
		A/A	52.38	48.28	1.179 (0.577–2.407)	0.204	>0.05
		G	27.80	30.20	0.890 (0.510–1.552)	0.168	>0.05
		A	72.20	69.80	1.123 (0.644–1.959)	0.168	>0.05
	680	G/G	3.17	6.90	0.443 (0.078–2.513)	0.888	>0.05
		G/A	47.61	37.93	1.488 (0.720–3.072)	1.157	>0.05
		A/A	49.22	55.17	0.787 (0.385–1.610)	0.431	>0.05
		G	27.00	25.90	1.059 (0.598–1.878)	0.039	>0.05
		A	73.0	74.10	0.944 (0.533–1.673)	0.039	>0.05
*INHBB*	365	C/C	69.84	77.59	0.669 (0.295–1.517)	0.931	>0.05
		C/A	30.16	22.41	1.495 (0.659–3.390)	0.931	>0.05
		A/A	0	0	–	–	–
		C	84.90	88.80	0.711 (0.334–1.513)	0.789	>0.05
		A	15.10	11.20	1.407 (0.661–2.995)	0.789	>0.05

GDF9, growth differentiation factor 9; BMP15, bone morphogenetic protein 15; *INHBB*, inhibin βB; FSHR, follicle stimulating hormone receptor; POF, premature ovarian failure; OR, odds ratio; CI, confidence interval.

**Table IV:** Comparison of the frequency of the *FSHR* 307 *GDF9* 546 combined genotype between patients with POF and control individuals.

*FSHR* 307-*GDF9* 546 combined genotype	Frequency in POF patients (*n*=63) n (%)	Frequency in controls (*n*=58) n (%)	OR (95% CI)	χ^2^-value	P-value
G/G-G/G	3 (4.76)	3 (5.17)	1.000 (reference)		
G/G-G/A	1 (1.59)	1 (1.72)	1.000 (0.041–24.547)	0.000	>0.05
G/A-A/A	–	–	–	–	–
G/A-G/A	5 (7.94)	9 (15.52)	1.800 (0.259–12.502)	0.010	>0.05
AnyA-G/G	41 (65.08)	43 (74.14)	1.049 (0.200–5.497)	0.000	>0.05
AnyA-G/A	17 (26.98)	9 (15.52)	0.529 (0.088–3.179)	0.055	>0.05
G/G-AnyA	1 (1.59)	1 (1.72)	1.000 (0.041–24.547)	0.000	>0.05
G/A-AnyA	8 (12.70)	4 (6.90)	0.500 (0.068–3.696)	0.029	>0.05
AnyA-AnyA	18 (28.57)	10 (17.24)	0.556 (0.094–3.285)	0.036	>0.05

GDF9, growth differentiation factor 9; FSHR, follicle stimulating hormone receptor; POF, premature ovarian failure; OR, odds ratio; CI, confidence interval.

**Table V:** Comparison of the frequency of the *FSHR* 680-*GDF* 546 combined genotype between patients with POF and controls.

*FSHR* 680-*GDF9* 546 combined genotype	Frequency in POF patients (*n*=63) n (%)	Frequency in controls (*n*=58) n (%)	Odds ratio (95% CI)	χ^2^-value	P-value
G/G-G/G	2 (3.17%)	1 (1.72)	1.000 (reference)		
G/G-G/A	2 (3.17%)	1 (1.72)	1.000 (0.034–29.807)	0.000	>0.05
G/A-A/A	–	–	–	–	–
G/A-G/A	9 (14.29%)	5 (8.62)	1.111 (0.079–15.534)	0.006	>0.05
AnyA-G/G	44 (69.84%)	45 (77.59)	2.045 (0.179–23.378)	0.000	>0.05
AnyA-G/A	16 (25.40%)	9 (15.52)	1.125 (0.089–14.202)	0.000	>0.05
G/G-AnyA	2 (3.17%)	1 (1.72)	1.000 (0.034–29.807)	0.000	>0.05
G/A-AnyA	9 (14.29%)	5 (8.62)	1.111 (0.079–15.534)	0.000	>0.05
AnyA-AnyA	17 (26.98%)	10 (17.24)	1.176 (0.094–14.686)	0.000	>0.05

GDF9, growth differentiation factor 9; FSHR, follicle stimulating hormone receptor; POF, premature ovarian failure; CI, confidence interval.

**Table VI:** Comparison of frequency of *FSHR* 307 680 combined genotype between patients with POF and controls.

*FSHR* 307-680 combined genotype	Frequency in POF patients (*n*=63) n (%)	Frequency in controls (*n*=58) n (%)	OR (95% CI)	χ^2^-value	P-value
G/G-G/G	2 (3.17%)	3 (5.17)	1.000 (reference)		
G/G-G/A	3 (4.76%)	–	–	–	–
G/A-A/A	2 (3.17%)	4 (6.90)	1.333 (0.113–15.704)	0	>0.05
G/A-G/A	23 (36.51%)	21 (36.21)	0.609 (0.092–4.007)	0.271	>0.05
AnyA-G/G	–	1 (1.72)	–	–	–
AnyA-G/A	27 (42.86%)	22 (37.93)	0.543 (0.083–3.545)	0.416	>0.05
G/G-AnyA	–	2 (3.45)	–	–	–
G/A-AnyA	2 (3.17%)	3 (5.17)	1.000 (0.08–12.557)	1.000	>0.05
AnyA-AnyA	58 (92.06%)	53 (91.38)	0.609 (0.098–3.788)	0.006	>0.05

FSHR, follicle stimulating hormone receptor; POF, premature ovarian failure; OR, odds ratio; CI, confidence interval.
